# Effect of Low versus Moderate Dose of Clofibrate on Serum Bilirubin in Healthy Term Neonates with Indirect Hyperbilirubinemia

**Published:** 2013-12

**Authors:** Fatemeh Eghbalian, Farnaz Monsef, Nazanin Alam Ghomi, Alireza Monsef

**Affiliations:** Department of Pediatrics, Bessat Hospital, Hamadan University of Medical Sciences, Hamadan, Iran

**Dear Editor,**

Clofibrate is an activator of peroxisome proliferators-activated receptors (PPARS). This agent has been used for decreasing lipid levels in adults for years.^1^^,^^2^ It is also a glucuronyl transferase inducer which may increase bilirubin conjunction and excretion and not only has a therapeutic effect on hyperbilirubinemia in term neonates but also prevents hyperbilirubinemia in preterm neonates.^3^ Other studies have demonstrated that a high dose of Clofibrate may lead to a reduction in both indirect bilirubin level and duration of hospitalization without known complications and side effects.^2^^-^^5^ Similar studies have evaluated the effect of a high dose of Clofibrate on neonatal hyperbilirubinemia. The aim of the present study was to compare the effects of a low dose (25 mg/kg) versus a moderate dose (50 mg/kg) of oral Clofibrate on the treatment of non-hemolytic hyperbilirubinemia in healthy term neonates. This single-blind, randomized, controlled clinical trial was approved by the Human Subject Review Board of Hamadan University of Medical Sciences. All the parents of the neonates recruited in this study signed informed written consent. The study population was comprised of 132 neonates with non-hemolytic indirect hyperbilirubinemia (total serum bilirubin [TSB]>16 mg/dl) admitted to the Neonatal Ward of Besat Hospital in the western Iranian city of Hamedan between November 2008 and June 2009. The sample size was calculated according to previous studies.^7^ The inclusion criteria consisted of age of 2 to 29 days; full-term birth (gestational age of between 38 to 40 weeks); weight of 2500 to 4000 gr; having indirect hyperbilirubinemia (TSB>16 mg/dl); absence of hemolysis, ABO, or Rh incompatibility; negative Coomb’s test; and reticulocyte count less than 5%. The exclusion criteria comprised signs of sepsis, electrolyte impairment, any congenital anomalies or diseases, seizure, formula feeding, hemolytic disease, and need for exchange transfusion. The selected neonates were allocated randomly (single blind) to three equal groups of 44 neonates: (1) control group, receiving only phototherapy; (2) intervention group I, receiving a single low dose of oral Clofibrate (25 mg/kg) plus phototherapy; and (3) intervention group II, receiving a single moderate dose of oral Clofibrate (50 mg/kg) plus phototherapy. Only the patients were kept blind to the type of treatment which they received. Clofibrate capsules of Zahravi Pharmaceutical Company, containing 500 mg Clofibrate, were dissolved in 5 cc distilled water. The calculated volume for each case was taken up with a syringe and was orally given to the patient. The control group did not receive any placebo. The three groups were matched for age, sex, birth weight, and gestational age. 

Total and indirect bilirubin levels were measured at the beginning of treatment and then 12, 24, 36, and 48 hours later. All the neonates were followed up for two days after discharge and were visited in our Outpatient Clinic for further evaluation of icterus and general health as well as probable Clofibrate adverse effects such as nausea, vomiting, urticaria, and anemia. TSB was measured using Pars Azmun Company biochemistry kits. Phototherapy was performed using five special blue lamps with wavelengths 420-450 nanometers (Tusan Company, Tehran-Iran). The independent samples t-test was employed for the comparison of bilirubin and the other parameters in the study. The data were analyzed with SPSS software (version 17). P values less than 0.05 were considered statistically significant. There was no significant difference in mean total bilirubin at baseline between the groups (P>0.05). There was a significant reduction in mean total bilirubin 12, 24, 36, and 48 hours after treatment in intervention groups I and II by comparison with the control group (P<0.001). However, there was no statistically significant difference between mean total bilirubin 12, 24, 36, and 48 hours after treatment between intervention group I and intervention group II (P<0.001). In this study, the neonates neither experienced any kind of adverse effect of Clofibrate nor needed exchange transfusion. Our findings in the present study are consistent with the results of other studies that have demonstrated the efficacy of Clofibrate in decreasing indirect hyperbilirubinemia and have also revealed that lower doses of Clofibrate can be used with the same therapeutic efficacy in reducing TSB levels in term infants with non-hemolytic hyperbilirubinemia.^3^^-^^6^ Lower, rather than higher, doses of Clofibrate can, therefore, be used to decrease TSB levels with lower side effects in healthy term neonates. In combination with phototherapy, Clofibrate (irrespective of its dosage) can reduce TSB levels in neonates with non-hemolytic indirect hyperbilirubinemia without adverse effects. A single dose of 25 mg/kg Clofibrate in the treatment of neonatal hyperbilirubinemia is effective and safe.
